# Dr. Robert A. Gunn

**Published:** 1913-01

**Authors:** 


Dr. Robert A. Gunn, Buffalo, 1866, of New York City, died of morphine poisoning in Bellevue Hospital, Dec. 7, aged 68.

